# Optimization of a Low-Power Chemoresistive Gas Sensor: Predictive Thermal Modelling and Mechanical Failure Analysis

**DOI:** 10.3390/s21030783

**Published:** 2021-01-25

**Authors:** Andrea Gaiardo, David Novel, Elia Scattolo, Michele Crivellari, Antonino Picciotto, Francesco Ficorella, Erica Iacob, Alessio Bucciarelli, Luisa Petti, Paolo Lugli, Alvise Bagolini

**Affiliations:** 1 MNF—The Micro Nano characterization and fabrication Facility, Bruno Kessler Foundation, Via Sommarive 18, 38123 Trento, Italy; novel@fbk.eu (D.N.); escattolo@fbk.eu (E.S.); crivella@fbk.eu (M.C.); picciotto@fbk.eu (A.P.); ficorella@fbk.eu (F.F.); iacob@fbk.eu (E.I.); 2 Faculty of Science and Technology, Free University of Bolzano-Bozen, Piazza Università 5, 39100 Bolzano, Italy; luisa.petti@unibz.it (L.P.); paolo.lugli@unibz.it (P.L.); 3 MST—MicroSystem Technology Group, Bruno Kessler Foundation, Via Sommarive 18, 38123 Trento, Italy; bucciarelli@fbk.eu

**Keywords:** silicon microheaters, chemoresistive gas sensors, predictive thermal model, mechanical failure analysis, response surface method

## Abstract

The substrate plays a key role in chemoresistive gas sensors. It acts as mechanical support for the sensing material, hosts the heating element and, also, aids the sensing material in signal transduction. In recent years, a significant improvement in the substrate production process has been achieved, thanks to the advances in micro- and nanofabrication for micro-electro-mechanical system (MEMS) technologies. In addition, the use of innovative materials and smaller low-power consumption silicon microheaters led to the development of high-performance gas sensors. Various heater layouts were investigated to optimize the temperature distribution on the membrane, and a suspended membrane configuration was exploited to avoid heat loss by conduction through the silicon bulk. However, there is a lack of comprehensive studies focused on predictive models for the optimization of the thermal and mechanical properties of a microheater. In this work, three microheater layouts in three membrane sizes were developed using the microfabrication process. The performance of these devices was evaluated to predict their thermal and mechanical behaviors by using both experimental and theoretical approaches. Finally, a statistical method was employed to cross-correlate the thermal predictive model and the mechanical failure analysis, aiming at microheater design optimization for gas-sensing applications.

## 1. Introduction

The detection of volatile compounds has become crucial for many different applications. Gas-sensing monitoring systems with high sensitivity are required for environmental monitoring, chemical process control, agriculture and medical applications [1,2]. Instruments mainly used for the accurate detection of gaseous compounds are based on light-matter interactions (IR and chemiluminescent spectroscopes) or on mass spectrometry [3,4,5,6]. Although these analyzers are particularly reliable, having both a high selectivity for the type of analyzed gas and low detection limits, their widespread sustainable use is still limited by significant shortcomings, including the high cost and the large size and weight that render them particularly cumbersome. 

In recent decades, huge attention has been paid to investigating and developing a new class of smart devices, i.e., solid-state gas sensors, which allow the monitoring of gas concentrations with ever-improving accuracy [7,8]. Among the various solid-state gas sensors investigated, the most studied are the chemoresistive gas sensors because of their versatility [9]. These devices are cost-effective, small, highly sensitive and suitable for portable instruments. The sensing layer is usually composed of a nanostructured semiconductor [9]. Many different nanomaterials were investigated and tested so far, with the aim of obtaining devices with even better sensing performances. Both hard [10,11] and soft [12,13] nanomaterials were exploited, as well as hybrid composites [14], towards improving sensitivity, selectivity and stability of the final sensing device. Usually, the nanostructured semiconductor needs to be thermal- or photo-activated to work properly, enabling a fast and reversible reaction between the gas target and sensing material surface [15,16,17,18]. Recently, the market demand for near-zero power consumption sensors, integrable in smartphones and other portable devices has driven the development of materials that work at room temperature, without any further activation [19]. For this purpose, different types of doping and functionalization have been applied to the semiconductors typically used as the sensing materials, enabling an increase of their surface reactivity and tunability of their electrical properties [19,20]. Furthermore, various innovative nanostructured materials and their composites have been tested. Among them, noteworthy are the 2D semiconductors (e.g., transition metal dichalcogenides and graphene), which highlight very interesting sensing performances due to their high area-to-volume ratio, high number of active sites and excellent electrical properties [20,21]. 

Although the advent of these innovative materials for chemoresistive gas sensors [22,23], Semiconducting Metal Oxide (SMO) still remain the most widely investigated and commercially used [24,25], employed in different applications [26,27,28,29,30]. These devices are composed of a nanostructured SMO as the sensing material and a heated substrate [9]. The role of the substrate is significant for the proper device operation, because it not only acts as a mechanical support of the sensing material but, also, it hosts the heater and electrodes. The former allows the heating of the sensing material at its best working temperature, whereas the latter are needed to read the electrical resistance of the sensing material [31]. The technical requirements for the optimal operation of a substrate are the high mechanical and thermal stability, low chemical reactivity, low power consumption and suitability for large-scale production. Until the 1990s, the most widely used material for substrate development was alumina, both for research and commercial purposes, due to its thermal and mechanical properties [32,33]. Nevertheless, its low processability, lack of chemical inertia and high thermal dissipation was the driving force for the research of more appropriate substrate materials [31,34]. Nowadays, silicon microheaters (MHs) are overwhelmingly diffused, primarily due to the high suitability of silicon for the microfabrication process and, in particular, for the development of technologies based on micro-electro-mechanical systems (MEMS). This well-established, reliable and high-throughput process allows for small, low-power consumption and cheap MHs [31,34,35]. Although the microfabrication process is well-known, great consideration needs to be devoted to designing the device, which strongly impacts MH performances [31,34,35,36]. In the standard configuration of a ready-to-use silicon MH, the heater and electrodes are placed over a free-standing membrane, obtained by silicon wet or dry etching, to avoid the thermal dissipation by conduction through the silicon bulk [31,34,35]. Therefore, a thermal and mechanical preliminary evaluation is suggested for the design of the optimal layout and microfabrication process for the MH development to obtain a device that meets the market demands of energy-saving and mechanical stability.

The possibility of producing low-consumption devices enables multiple applications where a continuous power supply through the power grid is not possible, such as air quality monitoring in remote locations or off-grid areas like nature reserves, where they could be used to check for leakage in pipes carrying hazardous gases. Different studies investigated the development of microheaters for gas-sensing purposes with ever-lower power consumptions. Hwang et al. developed a polysilicon microheater (heating area 2.3 × 2.6 mm^2^) showing a power consumption of 250 mW at 450 °C with the power consumption of 250 mW [37]. Several works have demonstrated that, by decreasing the heating area, a temperature of 400 °C can be reached with a power consumption of about 50 mW for silicon-based microheaters [38,39,40]. The most energy-efficient catalytic sensors showed a power consumption of about 15 mw at 450 °C [41], even if the introduction of innovative nanofabrication methodologies allows to further decrease that value.

Nevertheless, the estimation of the overall power consumption is challenging, and different approaches have been investigated so far [34,42]. To completely understand the heat transfer in a chemoresistive gas sensor, the following dissipation phenomena must be considered: (i) heat conduction, (ii) heat convection and (iii) radiation [43]. In free-standing membranes, the main contributions to heat dissipation are heat conduction along the membrane, heat conduction and heat convection by the surrounding air above and below the membrane. The radiation contribution can be considered negligible due to the relatively low working temperature [36,44,45,46]. The model proposed in this work aims at estimating the power consumption of a MH on a free-standing gas sensor membrane during the design phase, prior to the microfabrication process. The proposed model is strictly dependent on the geometrical features of the MH and the percentage area (A%) occupied by it on the membrane. Therefore, it provides a powerful tool for a MEMS heater design. A detailed discussion about the theoretical approach used for the model development will be presented. 

Alongside power consumption predictions [47], the failure of free-standing membranes is a crucial aspect to consider in device production. Failures can occur along the entire production process, from microfabrication to packaging and integration. Techniques such as reliability testing and a failure analysis are used to identify the root causes of failures and improve the MEMS design and production while guaranteeing a lower device infant mortality. The MIL-STD-883 procedure encompasses some of the highest requirements for reliability assessments of microcircuits used in space applications. Relevant assessments for free-standing membranes are mechanical tests. The standards include mechanical shock, visual inspections of components and vibration and bond testing (MIL-STD-883 method 2002.5, 2010.14, 2005.2, 2026, 2011.10 and 2023.7) [48]. However, in semiconductor manufacturing, reliability testing is mostly performed at the end of the production cycle, after the sensor is packaged or integrated with its electronics. Therefore, a more robust reliability assessment is required in the former production stages, i.e., during microfabrication. Thus, the need for identifying premature failures during microfabrication and the lack of a unified standard for such tests led scientists to the design of several setups for MEMS mechanical characterization [49,50,51]. The membrane deflection tests described in this study show a fast and versatile reliability testing by indentation. It can simulate the mechanical overload conditions that could develop, for instance, during the screen printing on silicon membrane arrays. The present setup employs modified bond test equipment with a design similar to that used by Fitzgerald et al. to apply torsion loads on MEMS micromirrors [52]. A clear advantage of this setup over bulge testing [49,50,51] is its compatibility with automated test methods. This setup offers fast screening for defective membranes and a qualification for the MEMS microfabrication process. Destructive indentation tests were performed on membranes with various lateral sizes. A statistical description of the failures of brittle membranes can be given with the Weibull model [53] to extract the correlation of the applied force at which each membrane fails and the likelihood of this happening. These data can be turned into a predictive model that is useful for comparing the mechanical strengths of ready-to-use MHs and setting the parameters of the sensor preparation process [31].

To summarize, in this work, three MH layouts with three different membrane areas were developed using the silicon microfabrication process. The performances of these devices were evaluated to predict their thermal and mechanical behaviors in an effort aimed at the optimization of the forthcoming MH layouts and process designs. A cross-correlation of the mechanical and thermal analyses provides a means to tune the design according to the required performance. 

## 2. Modeling of the Microheaters

### 2.1. Structure of the Microheaters

**Figure 1:**
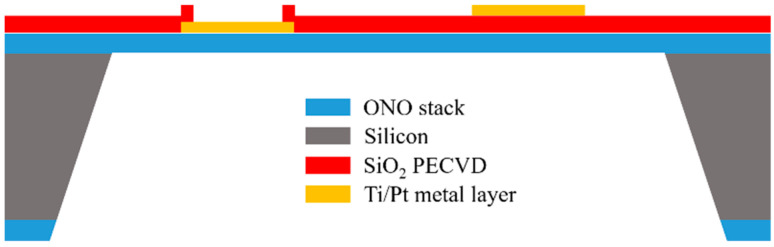
Cross-section of the silicon microheater (MH) produced in this work.

The fabrication process of silicon MHs used in this work was previously reported in [31]. The starting substrate was a double-sided polished p-type silicon wafer (thickness of 300 μm) with a <100> crystal orientation and a resistivity of 10–20 Ω·cm. A zero-stress stack called ONO, composed of SiO_2_/Si_3_N_4_/SiO_2_, was deposited over the silicon wafer by combining the thermal growth and low-pressure chemical vapor deposition (LPCVD). Afterwards, a stack consisting of titanium as an adhesion layer (10 nm) and platinum (120 nm) was deposited over the ONO stack by electron beam evaporation. Ti/Pt was used for both the heater and electrodes, and the layout was patterned by the lift-off technique. Platinum was chosen, because it provides excellent chemical stability and enables higher gas responses [54]. An intermetal dielectric layer was deposited by plasma-enhanced chemical vapor deposition (PECVD) to insulate the electrodes from the heater electrically. After each layer deposition, annealing at 650 °C in N_2_ for 2 h was carried out to thermally stabilize the deposited material. Finally, the membranes were released by wet etching, using a 25%-w tetramethylammonium hydroxide (TMAH) solution to remove the silicon. The size of each device, after the cutting step, was 3 × 3 mm^2^. Figure 1 shows the cross-section of the final devices. The equipment exploited in the microfabrication process is listed in the experimental section.

**Figure 2:**
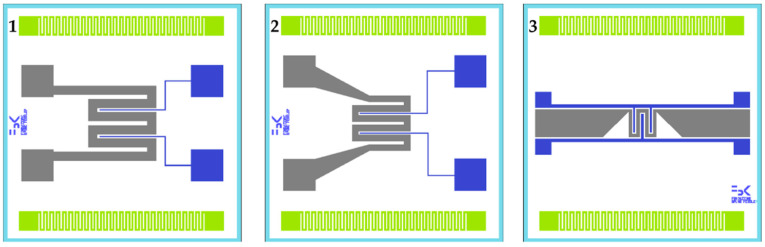
The three different layouts produced and tested in this work, named layouts 1, 2 and 3. In all the layouts, the heaters are represented by grey serpentines, while the electrodes by blue lines. Two reference resistances, depicted by green serpentines, were added in the devices for an extra evaluation of the temperature coefficient of resistance.

Three different layouts were produced and analyzed in this work, named layouts 1, 2 and 3 [55], as shown in Figure 2. 

**Figure 3:**
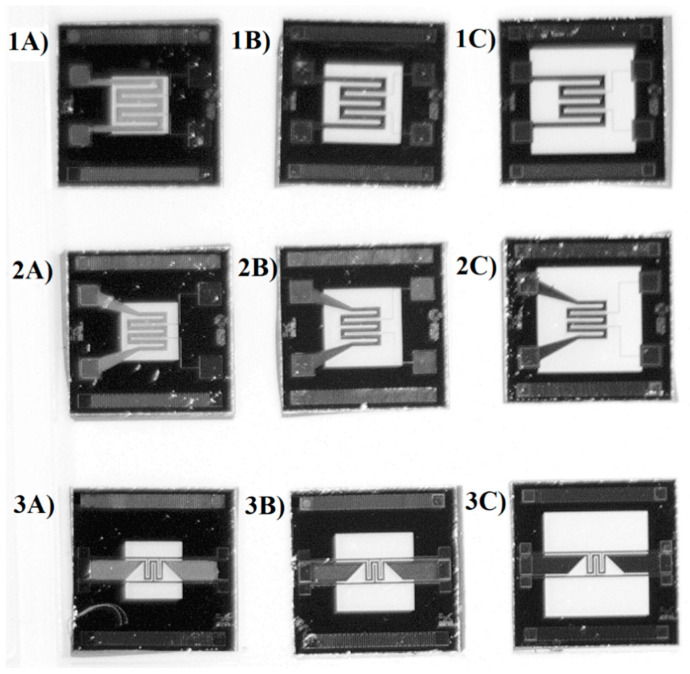
The three different membrane sizes developed and tested in this work for layouts 1–3.

For each of them, three different membrane areas were developed to investigate the influence of the membrane area on the mechanical and thermal performances of the MHs. The sizes of the three membranes were 0.9 × 0.9 mm^2^ (membrane A), 1.32 × 1.32 mm^2^ (membrane B) and 1.74 × 1.74 mm^2^ (membrane C), respectively (Figure 3).

### 2.2. Thermal Model

As discussed in the introduction, heat transfer in a free-standing membrane occurs mainly by heat conduction, heat convection and radiation. The contributions of conduction and radiation can be obtained experimentally by measuring the power consumption in a vacuum, removing the contribution of air dissipation, and by considering the different conduction (linear) and radiation (forth power) temperature dependencies [34]. This approach is effective for already available devices but can hardly be used to design new devices. In this work, based on the experimental results, simple models for basic understanding are developed. The total heat flow can be expressed as the sum of the different components of the thermal dissipation:

(1) $$Q_{tot}=G_{m}\;\lambda_{m}\;\left(T_{hot}-T_{room}\right)+G_{air}\;\lambda_{air}\;\left(T_{hot}-T_{room}\right)+G_{rad}\;\sigma \varepsilon \;\left(T_{hot}^{4}-T_{room}^{4}\right)$$

The above terms define the heat conduction through the closed membrane (first term), the heat conduction through the ambient air (second term) and the heat loss due to radiation (third term) in a steady-state condition. *G_m_*, *G_air_* and *G_rad_* are empirical values. They are geometry-related variables providing knowledge about the closed or suspended membrane geometry and its effects on heat loss. *T_hot_* and *T_room_* denote the temperatures of the hot active region and the ambient; the thermal conductivity of the membrane and the surrounding atmosphere are *λ_m_* and *λ_air_*, respectively; *ε* is the emissivity and *σ* the Stefan–Boltzmann constant. The various terms are discussed in the next sections, and expressions for the geometry factors are obtained.

#### 2.2.1. Membrane Heat Losses

**Figure 4:**
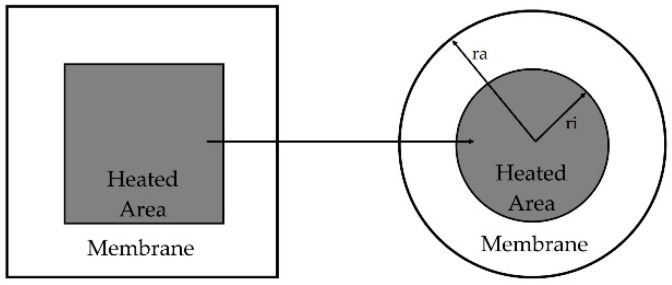
Schematic representation of a free-standing membrane in cylindrical coordinates. *r_i_* and *r_a_* are the radii of the heated area and the membrane, respectively.

For the evaluation of the membrane heat conduction, the perpendicular contribution is usually negligible compared to the parallel one. For a free-standing membrane, a simple model is obtained by approximating the square membrane as a round shape, therefore switching to cylindrical coordinates [34], as depicted in Figure 4. 

This leads to a one-dimensional heat conduction problem that can be easily solved by:

(2) $$Q_{mem}=\frac{2\Pi \lambda_{m}d\left(T_{hot}-T_{room}\right)}{ln\left(r_{a}/r_{i}\right)}$$

where *d* is the thickness of the membrane, *r_i_* and *r_a_* the radii of the heated area and the membrane, respectively, and *λ_mis_* the weighted average of an n-multilayer membrane.

#### 2.2.2. Air Heat Losses

Heat dissipation occurs by two processes, i.e., air convection and air conduction, as described above. With respect to the above, there are different contributions to the total heat dissipation of the top and bottom areas of the membrane. The air conduction contribution of the top area is given by:

(3) $$Q_{cond-top}=4\pi r_{i}\;\lambda_{air}\;\left(T_{hot}-T_{room}\right)$$

where *λ_air_* is the heat conduction coefficient of air as a function of the temperature. On the other hand, the contribution of the membrane bottom can be evaluated by:

(4) $$Q_{cond-bottom}=\frac{\lambda_{air}\;A\;\left(T_{hot}-T_{room}\right)\;}{h}$$

where *h* is the height of the etched silicon membrane, i.e., pit depth. 

Regarding the air convection, its evaluation is more complicated than that of the heat conduction. Given the lack of external forces, free convection is considered. The total heat flow (*Q_air_*) from a heated membrane area to the surrounding air can thus be described as:

(5) $$Q_{conv-air}=\alpha \left(m\right)A\;\left(T_{hot}-T_{room}\right)$$

where *α*(*m*) is the mean heat transfer coefficient. The theoretical determination of *α*(*m*) is rather difficult, requiring knowledge of the temperature and the air speed, and it is not reported in this work, because it has already been discussed in several manuscripts [20]. However, the heated free-standing membrane can be modeled as a horizontal plate of characteristic size l under the condition of free natural convection.

## 3. Materials and Methods

The MHs were developed in the clean rooms of the Bruno Kessler Foundation (FBK) [56]. A Centrotherm E 1200 HT 260-4 4 (Centrotherm International AG, Blaubeuren, Germany) diffusion and LPCVD furnaces were used for the thermal growth of SiO_2_ (nominal value 600 nm) and deposition of the Si_3_N_4_ (nominal value 100 nm) and SiO_2_ layers (nominal value 200 nm), with a total membrane thickness of about 1.0 μm. An ulvac EBX-16C with Ferrotec EV S-6 e-gun (ULVAC technologies, Inc., Kanagawa, Japan) was used for the deposition of the Ti and Pt layers. The thermal annealing of the silicon wafers was carried out using an Expertech CTR 200 (Expert Semiconductor Technology, Inc., Scotts Valley, CA, USA). The SiO_2_ intermetal passivation layer was deposited through an STS-MPS PECVD (STS Semiconductor and Telecommunications Co. Ltd., Seoul, Korea). SiO_2_ was etched over the pad areas of the heaters through an OEM Tegal 903 ACS reactive ion etcher (Tegal Corporation, Petaluma, CA, USA). Layouts of the deposited layers were defined by the photolithography technique, using an SVG 8600 Photoresist Coat Track (Silicon Valley Group, San Jose, CA, USA) and a Karl Suss automatic mask aligner (SUSS MicroTec Semiconductor, Garching, Germany).

An automatic prober Accretech UF200R (Accretech, Tokyo Seimitsu Co., Ltd., Tokyo, Japan) equipped with ATT LOW TEMP System L200T (Advanced Temperature Test Systems GmbH, Planegg, Germany) was used to perform the temperature coefficient of resistance (TCR) measurements on all the produced devices. This system provides a nominal temperature stability of ±0.1 °C, an accuracy of ±0.5 °C and a uniformity (along the chuck surface) less than 0.5%. Resistance measurements were performed at different temperatures (20 °C, 60 °C, 100 °C and 140 °C) to extract TCR parameters on heather and bulk resistors. Electrical measurements were performed using Agilent/Keysight equipment (Agilent Technologies, Santa Clara, CA, USA), including a low-leakage Switching Matrix Mainframe B2201A, with four modules of B2211A, and an SMU Mainframe E5270B, with four medium-power high-resolution SMU E5281B, two high-power high-resolution SMU 5280B and a ground unit. Both SMU types have a nominal resolution of 10 fA and 20 uV.

Manual measurements were performed using a Karl Suss Manual probing station PM8 (SUSS MicroTec Semiconductor, Garching, Germany), equipped with an Agilent 4156C Precision Semiconductor Parameter Analyzer (Agilent Technologies, Santa Clara, CA, USA) with a nominal resolution of 1 fA and 2 uV. Each measurement was repeated three times.

Tests were carried out to evaluate the resistance of different membrane sizes and layouts to the screen-printing pressure. The testing apparatus for destructive indentation tests was a shear/pull tester (Condor Sigma, XYZTEC, Panningen, The Netherlands) equipped with a stainless-steel flat conical tip with an end diameter of 290 µm and an axial load sensor of 20 N and high displacement sensitivity. The test procedure consisted of a manual approach along the z-axis, ending within 50 µm of distance from the surface of the membrane. The tip was optically aligned in the center of the membrane using heater geometry as the xy reference. The mechanical characterization was in a quasistatic regime with a vertical speed of 10 µm/s, and the data were recorded at a 250-Hz sampling frequency (or the highest sampling frequency). Test distance was set in the range of 150–400 µm to reach the fractures in all samples, depending on the deformability of the membranes. Sample fractures were video-recorded with a high-speed setting of 240 fps. The stainless-steel tip was cleaned from debris in between tests. Membrane testing was performed on silicon pieces containing arrays of several membranes. Each silicon piece was secured in place on a thick aluminum plate through a thin bi-adhesive Kapton tape. The sample size for each membrane set was the following: 20 MHs of 1A, 11 MHs of 1B, 30 MHs of 1C, 44 MHs of 2A, 11 MHs of 2B, 19 MHs of 2C, 8 MHs of 3A, 16 MHs of 3B and 30 MHs of 3C. The indentation tests were used to register the force–deflection curves until the fractures of the clamped square membranes. Each curve was analyzed to extract the MH failure force, defined as the maximum of the force–deflection curve, and the work of the fracture was defined as the integral of the curve.

A statistical method was used to cross-correlate the results obtained from the thermal predictive model and the mechanical failure analysis and to define a comprehensive model for tuning future sensor layouts to the required performance. The entire statistical analysis was done using the programming language R [57] following the statistical strategy described in previous works [58,59,60,61]. An initial comparison by verifying the presence of a significant difference among the various groups was performed by using the analysis of variance (ANOVA) and was followed by a Tukey multi-comparison test. The level of significance was assigned as follows: *p* ≤ 0.1 (.), *p* ≤ 0.05 (*), *p* ≤ 0.01 (**) and *p* ≤ 0.001 (***). A response surface methodology (RMS) was adopted to model the empirical equations relating the considered factors to the yields. In this case, we considered two continuous factors, the area covered by the heater (Factor *X*) and the length of the squared membrane (Factor *Y*), and two yields, the produced power (mW) and the mechanical strength (N). Table 1 shows the considered group. The complete model is reported in Equation (6). An ANOVA test followed by a Tukey multi-comparison test was conducted to verify the significance of each term of the reported equation. Only the terms with a significant effect (*p* ≥ 0.01) were included in the model. The function *F* was chosen to both normalize the model residues and to make them patternless. The model was considered significant with a *p*-value ≤ 0.05. To evaluate the goodness of the model fit, the coefficient of determination (r^2^) was calculated. A model with a perfect fit had a r^2^ = 1.

(6) $$F\left(Z\right)=c_{0}+c_{1}X+c_{2}Y+c_{3}XY+c_{4}X^{2}+c_{5}Y^{2}+c_{6}X^{2}Y+c_{7}XY^{2}+c_{8}X^{2}Y^{2}$$

## 4. Results and Discussion

### 4.1. Thermal Model for Power Consumption Evaluation

As discussed in Section 2.2, the analytic evaluation of the power consumption during the MH design phase is a challenging task. Here, we formalized a predictive model to establish the best A% (A% = MH area/membrane area). 

In order to simplify the approach, not all the contributions described in Section 2.2 were considered. Hille and Strack [42] hypothesized that the heat transfer from a free-standing membrane to the ambient is determined mainly by convective heat transfer on the top side and by thermal conduction through the air on the backside. They demonstrated that the constant of thermal conductivity (*λ_air_*) at the backside is three orders of magnitude lower than the heat transfer coefficient (*α*(*m*)) at the front side. Moreover, also, the irradiation contribution can be considered negligible due to the relatively low working temperature of the MH, which is about 400 °C. Therefore, in the model developed in this work, three thermal loss contributions were considered: (i) heat conduction along the membrane, (ii) heat convection from the top side and (iii) heat conduction through the air on the backside. Consequently, the overall power consumption is given by:

(7) $$Q_{tot}=Q_{mem}+Q_{top}+Q_{bot}$$

where *Q_mem_* is calculated in Equation (2), *Q_top_* in Equation (5) and *Q_bot_* in Equation (4).

To evaluate the effectiveness of the predictive thermal model, the theoretical total power consumption in Equation (7) is compared with the MH experimental power consumption on the three different membrane sizes: A—small, B—medium and C—large.

**Figure 5:**
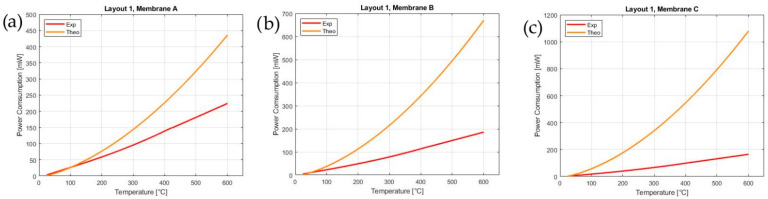
Comparison between the theoretical and experimental power consumptions of layout 1 over membranes A (**a**), B (**b**) and C (**c**).

Figure 5 displays the power consumption obtained using the first approximate model (Equation (7)). The model represented by Equation (7) does not fit the experimental data, predicting a much larger power consumption. The large mismatch is due to the incorrect initial hypothesis of the constant temperature of 400 °C over the entire membrane area. This shortcoming can be tackled with two different approaches: (i) via an experimental evaluation of the temperature gradient or (ii) by introducing a corrective coefficient (K).

Performing a precise in-situ experimental measurement of the temperature gradient can be challenging due to the limited spatial resolutions of the common infrared thermographic imaging techniques. Therefore, to outweigh the incorrect assumption of the constant working temperature on the entire membrane, we introduce the coefficient *K_EAD_*. The calculation of the corrective coefficient *K_EAD_* was based on the measured power consumption of layout 1 and then applied on layouts 2 and 3.

**Figure 6:**
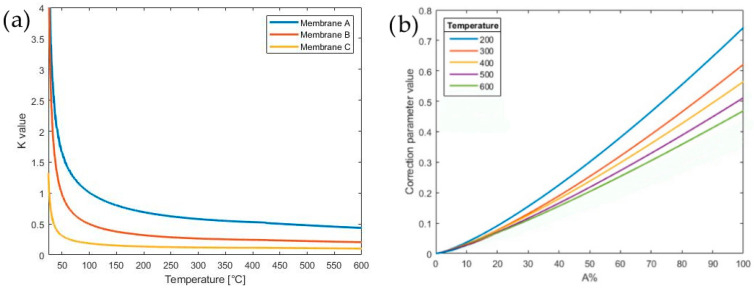
(**a**) The dependence of the coefficient K_EAD_ as function of the temperature, and (**b**) the dependence of the coefficient K_EAD_ as function of the A% occupied by the MH.

The equation to calculate the *K_EAD_* from the experimental results of layout 1 is given by:

(8) $$Q_{tot}=Q_{mem}+K_{EAD}\;\left(Q_{top}+Q_{bot}\right)$$

(9) $$K_{EAD}=\frac{Q_{top}-Q_{mem}}{Q_{top}+Q_{bot}}$$

where *Q_mem_*, *Q_top_* and *Q_bot_* were already explained in detail in Section 2.2. Figure 6a reports the value of the K_EAD_ calculated in Equation (8) as a function of the temperature and the membrane dimensions. It is clear that the value of *K_EAD_* is strongly affected by the temperature and by the A%. The plot shows an asymptotic value at a high temperature depending on the A%: the higher the A%, the higher the asymptotic value of *K_EAD_*.

Indeed, as shown in Figure 6b, the value of the correction coefficient *K_EAD_* can be modeled as a power law of A%, in which the coefficients are a function of the temperature, and it is given by:

(10) $$K_{EAD}\left(A\%,\;T\right)=a\left(T\right)\;A{\%}^{b\left(T\right)}$$

where the coefficients *a*(*T*) and *b*(*T*) are reported in Table 2. 

Thereby, using a corrective coefficient K_EAD_, it is possible to obtain an excellent fit of the thermal power consumption as a function of the working temperature. The developed predictive model was tested on layouts 2 and 3 for validation. The A% related to the three different layouts (1–3) and the three different membrane sizes (A–C) are listed in Table 3.

**Figure 7:**
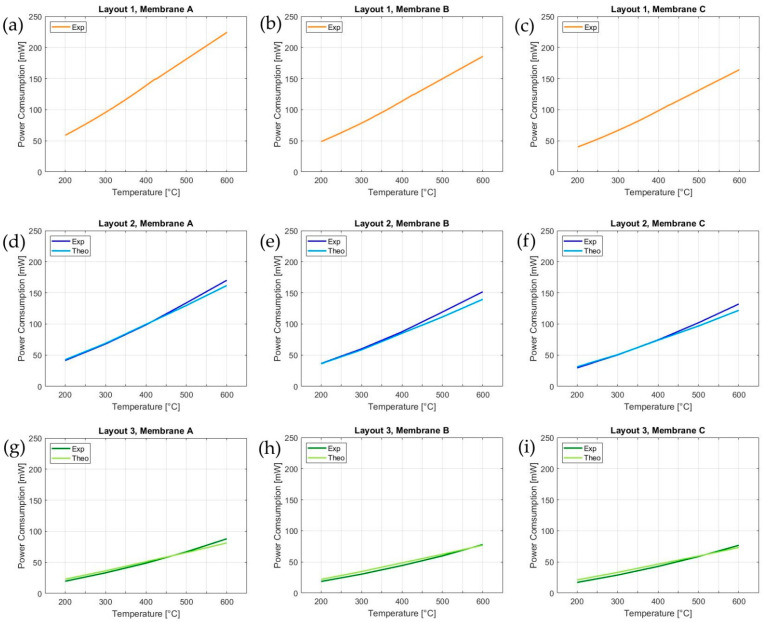
From (**a**–**c**) the experimental power consumption of layout 1 on membranes A–C, from (**d**–**f**) the comparison between the theoretical and experimental power consumptions of layout 2 on membranes A–C and from (**g**–**i**) the comparison between the theoretical and experimental power consumptions of layout 3 on membranes A–C.

The value of K_EAD_ of Figure 6 is calculated in the range 200–600 °C from the values of A% of layouts 2 and 3 reported in Table 3. Thereby, the theoretical power consumption of layouts 2 and 3 on membranes A, B and C are calculated and compared to the experimental measurements, as shown in Figure 7. As plotted in Figure 7, the predictive model with the constant K_EAD_ fits almost perfectly the experimental curves: this indicates that the model allows for the prediction of the power consumption of an MH during the design phase, achieving one of the purposes of this work. Clearly, the smaller the MH and the A% occupied, the lower the power consumption will be. Nevertheless, the results show that the membrane size significantly affects the power consumption only for A% higher than 50%. In conclusion, the results underline that the optimal A% of a MH is around 50%, since a lower A% does not significantly reduce the power consumption but negatively impacts the toughness of the membrane (Section 4.2).

The constant K_EAD_ and its analytic functions are related to the structure of the freestanding membrane and of the device investigated and produced in this work. Therefore, some adjustments must be made before applying the predictive thermal model to other devices.

### 4.2. Mechanical Failure Analysis 

The MH membrane should have high mechanical stability to withstand the stresses of the last microfabrication steps, such as cutting, deposition of the sensing material and bonding. In general, it should also be sufficiently robust to allow carefree handling of the final gas sensor and its use. Thus, an evaluation of the membrane mechanical stability is of utmost importance to define the quality of the working MHs and maximize the production yields of the sensors.

**Figure 8:**
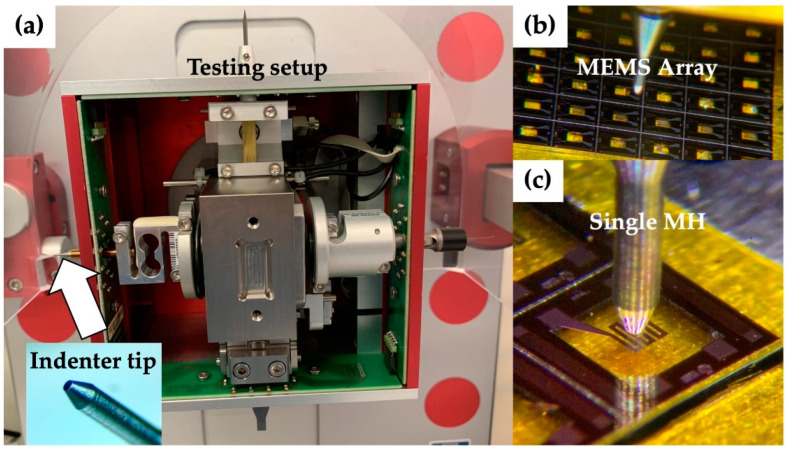
(**a**) Shear-pull tester adapted setup for axisymmetric indentation tests. (**b**) The modified setup can be used for high-throughput testing of the micro-electro-mechanical system (MEMS) array. (**c**) Indenter tip aligned for testing the MEMS MH (layout 1, membrane C).

Compared to bulge testing [48], where single membranes have to be handled singularly, indentation is a more appropriate reliability testing technique, as it enables the testing of large arrays (see Figure 8) with minimum delay between the test experiments and can be implemented and automated in a bond tester setup, as shown in this work.

**Figure 9:**
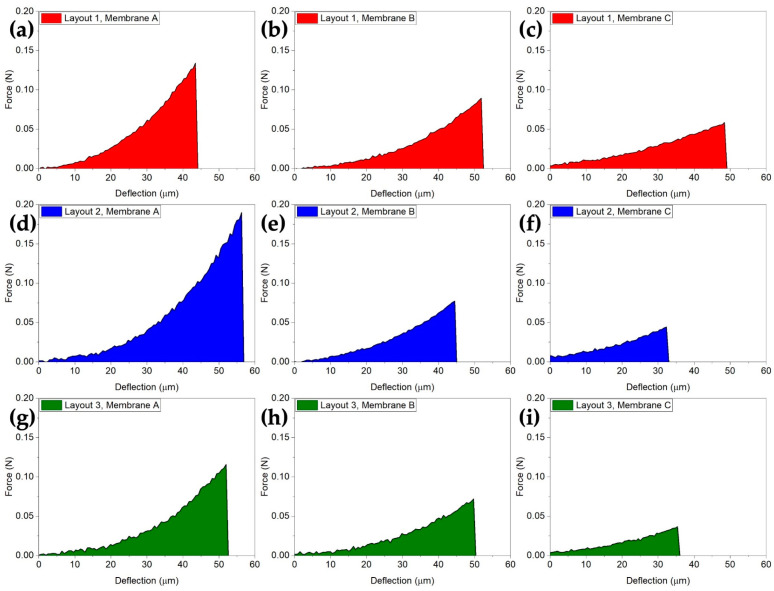
Average force–deflection curves from the destructive indentation testing. The shaded areas represent the work of the fractures. (**a**–**c**) Layout 1, (**d**–**f**) layout 2 and (**g**–**i**) layout 3. In each row, smaller-to-larger MH membranes are shown from left to right.

To date, a unified international standardization approach for testing the mechanical properties of MEMS [62] was lacking, while a wide variety of test methods were developed and used to characterize MEMS [49]. A rigid indenter setup offers the benefits of direct strain measurements compared to other methods [49,62], and it is suitable for automated testing. It allows for quick testing and the extraction of the characteristic force-deflection curves displayed in Figure 9.

Compared to bulge testing or axisymmetric tests on circular membranes, which can be modeled with a closed analytical formulation, the large deformations reached in destructive indentation tests on rectangular-shaped membranes often require a more complex formulation with a higher number of parameters to be solved [51,63] or a finite element analysis [51,53,64] to obtain an accurate representation of a multilayer MEMS.

**Figure 10:**
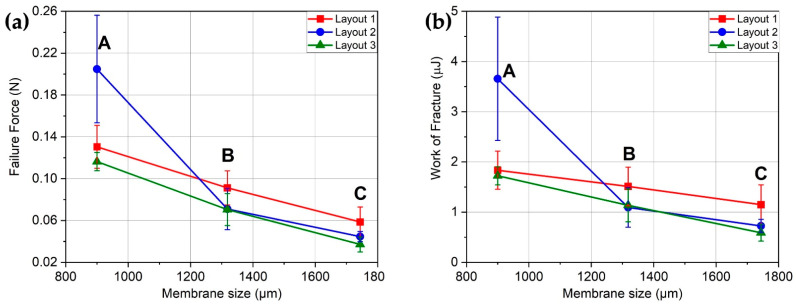
Indentation tests analysis. (**a**) Failure forces and (**b**) work to fracture of the different sets of MHs. Both show a downward trend. Average values and standard deviation are displayed.

The force–deflection curves of Figure 9 are correlated with the mechanical stability and resistance of MH throughout the sensor fabrication process. For instance, the failure force (see Figure 10a) gives a useful reference for the maximum force that MH can withstand. A similar indication (see Figure 10b) can be found in the work to fracture required to break the MH having dimensions in the range of µJ. Comparing different layout and sizes, we report a five ÷ six-fold difference between the strongest and toughest layout (2A), with 205 mN of failure force and 3.66 µJ of work to fracture, and the least-resistant layout (3C) that showed 37 mN and 0.59 µJ. The overall trend of the three membrane layouts was downward in both the failure force and work to fracture by increasing the membrane size from A to B and C. For all three layouts, the small membranes were the strongest. This is expected behavior due to the defect–size effect that makes smaller membranes (A) more resistant.

To sum up, these data are valuable not only as a qualification test or to guide design choices but, also, to understand how many membranes will break during the successive processing steps.

Among the steps inducing stress on the produced membranes, the sensing material deposition generates one of the most demanding loading conditions. The deposition ranges from in-situ self-assembly [65] to inkjet printing [66] and screen printing [67], which are ranked by increasing the applied force on the substrate. Particularly for screen-printing, which is the technique mainly used to produce SMO thick-film gas sensors, MEMS damage is not rare. Thus, a common prerequisite in the literature is to use the lowest force to minimize the membrane damage [68].

However, this indication that lacks the force ranges is not enough for the best design practices, so a direct mechanical characterization is required to cover this gap. The objective of the destructive deflection tests of this work is to provide a model for predicting the membrane failures that consider multiple parameters, such as the membrane size, layout and statistical distribution of the membrane strengths and the pressure applied during screen printing of the sensor film.

Destructive testing prior to sensor film deposition is required to determine the scale and shape parameters of the statistical distribution of membrane strength for each layout. A Weibull two-parameters fit was chosen, as it well-describes [53] the failure distributions of brittle ONO membranes. The probability density function describes the likelihood of membrane failures in the following equation:

(11) $$PDF=\frac{b}{a}{\left(\frac{x}{a}\right)}^{b-1}e^{-{\left(x/a\right)}^{b}}$$

where *x* is the measured failure force of MH, and *a* and *b* are the scale and shape parameters of the Weibull distribution, respectively. This statistical distribution is used as the basis for the prediction of failure, as represented in Figure 9. Indeed, it is possible to predict the yield in terms of a broken MH after the experimental assessment of the minimum squeegee force needed for a good quality deposition of the sensor film (i.e., 0.16 N on a 3 × 3 mm^2^ device) and the knowledge of the probability density function for MH failures.

The force acting on a single membrane is calculated from the area fraction that each membrane occupies on the wafer and with a squeegee applying an equally distributed pressure on the array of silicon devices. Thus, the cumulative probability of failures for an MH membrane at a given applied force *x* is:

(12) $$p=F\left(x|a,b\right)=\int_{0}^{x}ba^{-b}t^{b-1}e^{-{\left(\frac{t}{a}\right)}^{b}}$$

These distributions can be used in practice. Using as input the force acting on the single MH, which is a fraction of the 0.16-N deposition force, the percentage of broken MH yielded from the deposition process can be estimated, as shown in Table 4.

**Figure 11:**
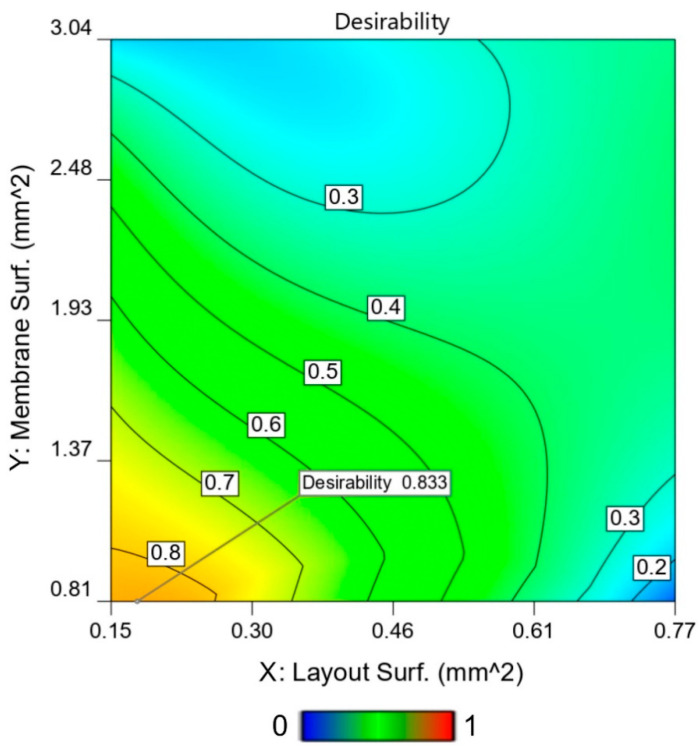
Contour plot of the calculated total desirability. The numerical system was optimized to both minimize the power consumption and to maximize the mechanical strength. The optimal result is signed in the graph with a flag. Interestingly, the optimal point result was between the tested points, indicating the fact that the resolution of the problem was nontrivial.

We notice that the membrane size C shows an estimated percentage of broken membranes that is too high to be acceptable for production. Indeed, the desirability of the three sets of the C membrane is the lowest (see Figure 11) and is not advised as the support structure for chemoresistive gas sensing. Therefore, for design purposes, only A and B sizes should be considered. Both sets show a very high production yield, with a maximum percentage of about 2% of broken MHs. Regarding the mechanical stability, both A and B membrane sizes can be used in the MH for gas sensor development.

### 4.3. Statistical Model for the Cross-Correlation of Thermal and Mechanical Analyses

A statistical method was employed to cross-correlate the thermal predictive model and mechanical failure analysis. The cross-correlation aims to define a comprehensive empirical model for the optimization of the MH design. 

First, two separated empirical models were developed for power consumption and mechanical response by following the approach reported in the experimental section. The empirical model for power consumption (Equation (13)) fits the data with a *r*^2^ = 0.998, whereas the one developed for the mechanical response (Equation (14)) had a *r*^2^ = 0.924.

(13) $$\begin{array}{r} P\left(mW\right)=36.199+166.749X-17.2561Y+33.9073XY-61.5141X^{2}+\\ 4.75602Y^{2}-51.2679X^{2}Y-18.8218XY^{2}+20.6892X^{2}Y^{2} \end{array}$$

(14) $$\begin{array}{r} F\left(N\right)=-3.71972+16.44X+1.44057Y-16.5341XY-17.9295X^{2}-\\ 0.422435Y^{2}+18.2888X^{2}Y+3.6002XY^{2}-3.92455X^{2}Y^{2} \end{array}$$

Then, the merge and optimization of the empirical models was performed via a numerical method based on the desirability functions. These functions are in the [1, 0] range, where 1 represents the optimum solution. One of these functions was assigned to each of the considered yields. We used the following notation: *Z_i_* as the specific yield, di as the corresponding desirability function and *U_i_* and *Li* as the maximum and the minimum values of the yield, respectively. For the maximization of *Z_i_*, the function is reported in Equation (15), and, for the minimization, in Equation (16). The overall desirability is the geometric mean of all these functions, as reported in Equation (17), with k equal to the total number of yields (in our case, two). Then, *D* is plotted against the process factors to find its minimum value and, thus, the best solution.

(15) $$d_{i}=\{\begin{array}{ll} 1 & if\;Z_{i}\geq U_{i}\\ \frac{Z_{i}-L_{i}}{U_{i}-L_{i}} & if\;L_{i}\geq Z_{i}\geq U_{i}\\ 0 & if\;Z_{i}\leq L_{i} \end{array}$$

(16) $$d_{i}=\{\begin{array}{ll} 0 & if\;Z_{i}\geq U_{i}\\ \frac{Z_{i}-L_{i}}{U_{i}-L_{i}} & if\;L_{i}\geq Z_{i}\geq U_{i}\\ 1 & if\;Z_{i}\leq L_{i} \end{array}$$

(17) $$D={\left(d_{1}d_{2}d_{3}\ldots d_{k}\right)}^{\frac{1}{k}}$$

A specific case study was investigated by exploiting the desirability factor. Aiming at the design optimization for future MH development, it was decided to consider the maximization of the mechanical response (fracture force) while minimizing the power consumption. Furthermore, the maximum force applied to the MH during the screen-printing deposition (0.16 N) was chosen as a threshold for the mechanical response. Indeed, MHs with a mechanical performance above this maximum threshold are welcome, even if they do not offer a significantly desirable advantage for a MH design.

The color-coded desirability obtained is reported in Figure 11.

The desirability trend obtained in this specific case study is in-line with the standard physical interpretation. Giving the same importance to the two yields, we discovered that the highest desirability is obtained by increasing as much as possible the mechanical stability of the membrane (which is obtained for smaller membranes, as shown in Section 4.2) and decreasing the power consumption of the MH, which decreases by decreasing the heater layout surface (Section 4.1). Interestingly, the highest desirability solution is not trivial, depicted in Figure 11 (desirability of 0.833), as it lies outside of the tested points but very close to layout 3 on the smallest membrane area produced (A). However, the trend for increasing the desirability in the tested domain is clear, and it might be possible to achieve a higher desirability with a bigger layout and smaller membrane size than the 3A MH. In conclusion, the desirability surface lines obtained in Figure 11 are beneficial for the production of forthcoming microheaters. They allow us to identify the best compromise between the type of layout and membrane size in order to obtain a MH with low power consumption and high mechanical stability. Indeed, silicon-based gas sensors are devices that require power efficiency, sensing abilities and mechanical stability during fabrication and during operation to withstand high thermal stresses [69]. Thereby, to guide design choices, an approach that couples all the previous factors should be considered, as it could lead to the highest desirability [70]. Being able to tune beforehand the power consumption is useful for outdoor applications [71], where the battery life represents a cap on the performance, or smartphone portable air monitoring systems [72] and self-powered wearable devices [73]. 

The empirical model here reported can be further expanded and improved by considering other variables, such as the uniformity of the temperature distribution on the MH and the mechanical stability of the membrane at high temperatures. 

## 5. Conclusions

In this work, three different microheater layouts on three different membrane areas were produced at the FBK by exploiting silicon microfabrication technology. To optimize future MH layouts and process designs, the thermal and mechanical efficiencies of these devices were assessed by using both experimental and theoretical methods to predict their behaviors. In particular, the predictive thermal model was developed to correlate the power consumption and a design parameter of the MH called A% (i.e., the percentage of the membrane area occupied by the heater). The model considered three contributions: (i) heat conduction along the membrane, (ii) heat convection from the top side and (iii) heat conduction through the air on the backside. A dedicated coefficient (K_EAD_), evaluated from the experimental characterization of the MH thermal performance, was also introduced into the model as a correction from the initial ideal assumption of a constant working temperature over the entire membrane. The final model matched the experimental data very well and illustrated the close relationship between A% and the power consumption. The failure analysis showed that the membrane size and MH layout greatly impact the failure forces, with the smaller membranes being the strongest. Furthermore, the analysis led to an estimation of the yield in terms of working devices from the final processing steps. In conclusion, a statistical method was used to combine the results of the thermal and mechanical investigations. A desirability factor was introduced for the predictive evaluation of MH performances, a new tool to define the best combination of layouts and membrane areas to design forthcoming microheaters for gas-sensing applications.

## Figures and Tables

**Table 1:** Combinations of membranes and layouts tested with the corresponding associated continuous factors. The values of the factors were used to build the empirical models during the statistical analysis.

Group	Factor *X*-Area Layout (mm^2^)	Factor *Y*-Area Membrane (mm^2^)
1	0.15	0.81
2	0.15	1.74
3	0.15	3.01
4	0.56	0.81
5	0.56	1.74
6	0.56	3.01
7	0.77	0.81
8	0.77	1.74
9	0.77	3.01

**Table 2:** Values of the coefficients *a*(*T*) and *b*(*T*) in the temperature range of 200–600 °C.

*T* (°C)	*a*(*T*)	*b*(*T*)
200	0.001839	1.303
300	0.001590	1.296
400	0.001794	1.249
500	0.001742	1.234
600	0.001825	1.213

**Table 3:** Values of A% occupied by layouts 1–3 over membranes A–C.

Membrane	Layout 1	Layout 2	Layout 3
A	95%	69%	18%
B	44%	32%	9%
C	25%	18%	5%

**Table 4:** Estimated percentage of the broken microheater (MH) for each set of membranes. The values were derived according to a vertical deposition force of 0.16 N equally distributed over each device surface.

Membrane Layout	Membrane Size	Estimate Percentage of Broken MH (%)
1	A	~0
1	B	~0
1	C	37
2	A	~0
2	B	2
2	C	100
3	A	~0
3	B	1
3	C	100

## Data Availability

The data that support the findings of this study are available from the corresponding authors upon request.

## References

[1] Santer B.D., Taylor K.E., Wigley T.M.L., Penner J.E., Jones P.D., Cubasch U. (1995). Towards the detection and attribution of an anthropogenic effect on climate. Clim. Dyn..

[2] Soloman S. (2009). Sensors Handbook.

[3] Kind T., Fiehn O. (2010). Advances in structure elucidation of small molecules using mass spectrometry. Bioanal. Rev..

[4] De Gouw J., Warneke C. (2007). Measurements of volatile organic compounds in the earth’s atmosphere using proton-transfer-reaction mass spectrometry. Mass Spectrom. Rev..

[5] Isacsson U., Wettermark G. (1974). Chemiluminescence in analytical chemistry. Anal. Chim. Acta.

[6] Werle P., Slemr F., Maurer K., Kormann R., Mücke R., Jänker B. (2002). Near- and mid-infrared laser-optical sensors for gas analysis. Opt. Laser Eng..

[7] Comini Elisabetta Faglia G., Sberveglieri G. (2008). Solid State Gas Sensing.

[8] Hunter G.W., Akbar S., Bhansali S., Daniele M., Erb P.D., Johnson K., Liu C.-C., Miller D., Oralkan O., Hesketh P.J. (2020). Editors′ Choice-Critical Review—A Critical Review of Solid State Gas Sensors. J. Electrochem. Soc..

[9] Neri G. (2015). First fifty years of chemoresistive gas sensors. Chemosensors.

[10] Rackauskas S., Barbero N., Barolo C., Viscardi G. (2017). ZnO nanowire application in chemoresistive sensing: A review. Nanomaterials.

[11] Neri G., Leonardi S.G., Latino M., Donato N., Baek S., Conte D.E., Russo P.A., Pinna N. (2013). Sensing behavior of SnO_2_/reduced graphene oxide nanocomposites toward NO_2_. Sens. Actuators B Chem..

[12] Kumar V., Mirzaei A., Bonyani M., Kim K.-H., Kim H.W., Kim S.S. (2020). Advances in electrospun nanofiber fabrication for polyaniline (PANI)-based chemoresistive sensors for gaseous ammonia. Trends Analyt. Chem..

[13] Bertoni C., Naclerio P., Viviani E., Dal Zilio S., Carrato S., Fraleoni-Morgera A. (2019). Nanostructured p3ht as a promising sensing element for real-time, dynamic detection of gaseous acetone. Sensors.

[14] Willa C., Yuan J., Niederberger M., Koziej D. (2015). When nanoparticles meet poly(ionic liquid)s: Chemoresistive CO_2_ sensing at room temperature. Adv. Funct. Mater..

[15] Gaiardo A., Fabbri B., Giberti A., Guidi V., Bellutti P., Malagù C., Valt M., Pepponi G., Gherardi S., Zonta G. (2016). ZnO and Au/ZnO thin films: Room-temperature chemoresistive properties for gas sensing applications. Sens. Actuators B Chem..

[16] Gaiardo A., Fabbri B., Guidi V., Bellutti P., Giberti A., Gherardi S., Vanzetti L., Malagù C., Zonta G. (2016). Metal sulfides as sensing materials for chemoresistive gas sensors. Sensors.

[17] Giberti A., Fabbri B., Gaiardo A., Guidi V., Malagù C. (2014). Resonant photoactivation of cadmium sulfide and its effect on the surface chemical activity. Appl. Phys. Lett..

[18] Zonta G., Astolfi M., Casotti D., Cruciani G., Fabbri B., Gaiardo A., Gherardi S., Guidi V., Landini N., Valt M. (2020). Reproducibility tests with zinc oxide thick-film sensors. Ceram. Int..

[19] Srinivasan P., Ezhilan M., Kulandaisamy A.J., Babu K.J., Rayappan J.B.B. (2019). Room temperature chemiresistive gas sensors: Challenges and strategies—A mini review. J. Mater. Sci. Mater. Electron..

[20] Joshi N., Hayasaka T., Liu Y., Liu H., Oliveira O.N., Lin L. (2018). A review on chemiresistive room temperature gas sensors based on metal oxide nanostructures, graphene and 2D transition metal dichalcogenide. Microchim. Acta.

[21] Hashtroudi H., MacKinnon I.D.R., Shafiei M. (2020). Emerging 2D hybrid nanomaterials: Towards enhanced sensitive and selective conductometric gas sensors at room temperature. J. Mater. Chem. C.

[22] Gaiardo A., Fabbri B., Giberti A., Valt M., Gherardi S., Guidi V., Malagù C., Bellutti P., Pepponi G., Casotti D. (2020). Tunable formation of nanostructured SiC/SiOC core-shell for selective detection of SO_2_. Sens. Actuators B Chem..

[23] Guidi V., Fabbri B., Gaiardo A., Gherardi S., Giberti A., Malagù C., Zonta G., Bellutti P. (2015). Metal sulfides as a new class of sensing materials. Procedia. Eng..

[24] Dey A. (2018). Semiconductor metal oxide gas sensors: A review. Mat. Sci. Eng. B Adv..

[25] Degler D. (2018). Trends and advances in the characterization of gas sensing materials based on semiconducting oxides. Sensors.

[26] Fabbri B., Valt M., Parretta C., Gherardi S., Gaiardo A., Malagù C., Mantovani F., Strati V., Guidi V. (2020). Correlation of gaseous emissions to water stress in tomato and maize crops: From field to laboratory and back. Sens. Actuators B Chem..

[27] Zonta G., Anania G., Astolfi M., Feo C., Gaiardo A., Gherardi S., Giberti A., Guidi V., Landini N., Palmonari C. (2019). Chemoresistive sensors for colorectal cancer preventive screening through fecal odor: Double-blind approach. Sens. Actuators B Chem..

[28] Zonta G., Anania G., de Togni A., Gaiardo A., Gherardi S., Giberti A., Guidi V., Landini N., Palmonari C., Ricci L. (2018). Use of Gas Sensors and FOBT for the Early Detection of Colorectal Cancer. Sens. Actuators B Chem..

[29] Piedrahita R., Xiang Y., Masson N., Ortega J., Collier A., Jiang Y., Li K., Dick R.P., Lv Q., Hannigan M. (2014). The next generation of low-cost personal air quality sensors for quantitative exposure monitoring. Atmos. Meas. Tech..

[30] Gaiardo A., Zonta G., Gherardi S., Malagù C., Fabbri B., Valt M., Vanzetti L., Landini N., Casotti D., Cruciani G. (2020). Nanostructured SmFeO_3_ gas sensors: Investigation of the gas sensing performance reproducibility for colorectal cancer screening. Sensors.

[31] Bagolini A., Gaiardo A., Crivellari M., Demenev E., Bartali R., Picciotto A., Valt M., Ficorella F., Guidi V., Bellutti P. (2019). Development of MEMS MOS gas sensors with CMOS compatible PECVD inter-metal passivation. Sens. Actuators B Chem..

[32] Ochiwa Shinichi (Fuji Electric Co Ltd.) (1991). Gas Sensor. Japanese Patent.

[33] Martinelli G., Carotta M.C., Ferroni M., Sadaoka Y., Traversa E. (1999). Screen-printed perovskite-type thick films as gas sensors for environmental monitoring. Sens. Actuators B Chem..

[34] Simon I., Bârsan N., Bauer M., Weimar U. (2001). Micromachined metal oxide gas sensors: Opportunities to improve sensor performance. Sens. Actuators B Chem..

[35] Ma H., Du Y., Wei M., Ding E., Lin L. (2019). Silicon microheater based low-power full-range methane sensing device. Sens. Actuators A.

[36] Lahlalia A., Filipovic L., Selberherr S. (2018). Modeling and Simulation of Novel Semiconducting Metal Oxide Gas Sensors for Wearable Devices. IEEE Sens. J..

[37] Hwang W.-J., Shin K.-S., Roh J.-H., Lee D.-S., Choa S.-H. (2011). Development of micro-heaters with optimized temperature compensation design for gas sensors. Sensors.

[38] Belmonte J.C., Puigcorbé J., Arbiol J., Vilà A., Morante J.R., Sabaté N., Gràcia I., Cané C. (2006). High-temperature low-power performing micromachined suspended micro-hotplate for gas sensing applications. Sens. Actuators B Chem..

[39] Bhattacharyya P. (2014). Technological journey towards reliable microheater development for MEMS gas sensors: A review. IEEE Trans. Device Mat. Reliab..

[40] Prajesh R., Jain N., Agarwal A. (2016). Low power highly sensitive platform for gas sensing application. Microsyst. Technol..

[41] Baranov A., Spirjakin D., Akbari S., Somov A. (2015). Optimization of power consumption for gas sensor nodes: A survey. Sens. Actuator A Phys..

[42] Hille P., Strack H. (1992). A heated membrane for a capacitive gas sensor. Sens. Actuators A.

[43] Sberveglieri G., Hellmich W., Müller G. (1997). Silicon hotplates for metal oxide gas sensor elements. Microsyst. Technol..

[44] Iwaki T., Covington J.A., Udrea F., Ali S.Z., Guha P.K., Gardner J.W. (2005). Design and simulation of resistive SOI CMOS micro-heaters for high temperature gas sensors. J. Phys. Conf. Ser..

[45] Lee S.M., Dyer D.C., Gardner J.W. (2003). Design and optimisation of a high-temperature silicon micro-hotplate for nanoporous palladium pellistors. Microelectron. Eng..

[46] Fung S.K.H., Tang Z., Chan P.C.H., Sin J.K.O., Cheung P.W. (1996). Thermal analysis and design of a micro-hotplate for integrated gas-sensor applications. Sens. Actuators A.

[47] Zhang K.L., Chou S.K., Ang S.S. (2007). Fabrication, modeling and testing of a thin film Au/Ti microheater. Int. J. Therm. Sci..

[48] USDOD (2017). Test Method Standard: Microcircuits, Military Standard 883K.

[49] Srikar V.T., Spearing S.M. (2003). A critical review of microscale mechanical testing methods used in the design of microelectromechanical systems. Exp. Mech..

[50] Vlassak J.J., Nix W.D. (1992). A new bulge test technique for the determination of Young’s modulus and Poisson’s ratio of thin films. J. Mater. Res..

[51] Gaspar J., Ruther P., Paul O. (2006). Mechanical characterization of thin-film composites using the load-deflection response of multilayer membranes-elastic and fracture properties. Mater. Res. Soc. Symp. Proc..

[52] Fitzgerald A.M., Pierce D.M., Benedikt Z. (2010). “Predicting reliability of silicon MEMS.” Reliability, Packaging, Testing, and Characterization of MEMS/MOEMS and Nanodevices IX.

[53] Jadaan O.M., Nemeth N.N., Bagdahn J., Sharpe W.N. (2003). Probabilistic Weibull behavior and mechanical properties of MEMS brittle materials. J. Mater. Sci..

[54] Barsan N., Schweizer-Berberich M., Göpel W. (1999). Fundamental and practical aspects in the design of nanoscaled SnO_2_ gas sensors: A status report. Fresenius J. Anal. Chem..

[55] Vasiliev A.A., Pisliakov A.V., Sokolov A.V., Polovko O.V., Samotaev N.N., Kujawski W., Rozicka A., Guarnieri V., Lorencelli L. (2014). Gas sensor system for the determination of methane in water. Procedia. Eng..

[56] https://cmm.fbk.eu/en/research/mnf-micro-nano-facility/.

[57] R Core Team (2019). R: A Language and Environment for Statistical Computing.

[58] Bucciarelli A., Reddy Chandraiahgari C., Adami A., Mulloni V., Lorenzelli L. (2020). Precise Dot Inkjet Printing Thought Multifactorial Statistical Optimization of the Piezoelectric Actuator Waveform. Flex. Print. Electron..

[59] Bucciarelli A., Muthukumar T., Kim J.S., Kim W.K., Quaranta A., Maniglio D., Khang G., Motta A. (2019). Preparation and Statistical Characterization of Tunable Porous Sponge Scaffolds Using UV Cross-Linking of Methacrylate-Modified Silk Fibroin. ACS Biomater. Sci. Eng..

[60] Bucciarelli A., Chiera S., Quaranta A., Yadavalli V.K., Motta A., Maniglio D. (2019). A Thermal-Reflow-Based Low-Temperature, High-Pressure Sintering of Lyophilized Silk Fibroin for the Fast Fabrication of Biosubstrates. Adv. Funct. Mater..

[61] Bucciarelli A., Adami A., Chandaiahgari C.R., Lorenzelli L. Multivariable Optimization of Inkjet Printing Process of Ag Nanoparticle Ink on Kapton. Proceedings of the 2020 IEEE International Conference on Flexible and Printable Sensors and Systems (FLEPS).

[62] Edwards R.L., Coles G., Sharpe W.N. (2004). Comparison of tensile and bulge tests for thin-film silicon nitride. Exp. Mech..

[63] Carrera E., Ciuffreda A. (2005). A unified formulation to assess theories of multilayered plates for various bending problems. Compos. Struct..

[64] Hussein Al-Tholaia M.M., Al-Gahtani H.J. (2016). RBF-Based Meshless Method for Large Deflection of Elastic Thin Rectangular Plates with Boundary Conditions Involving Free Edges. Math. Probl. Eng..

[65] Dai Z., Xu L., Duan G., Li T., Zhang H., Li Y., Cai W. (2013). Fast-response, sensitivitive and low-powered chemosensors by fusing nanostructured porous thin film and IDEs-microheater chip. Sci. Rep..

[66] Walden P., Kneer J., Knobelspies S., Kronast W., Mescheder U., Palzer S. (2015). Micromachined hotplate platform for the investigation of ink-jet printed, functionalized metal oxide nanoparticles. J. Microelectromech. Syst..

[67] Viricelle J.P., Rivière B., Pijolat C. (2005). Optimization of SnO_2_ screen-printing inks for gas sensor applications. J. Eur. Ceram. Soc..

[68] Vincenzi D., Butturi M.A., Guidi V., Carotta M.C., Martinelli G., Guarnieri V., Pignatel G.U. (2001). Development of a low-power thick-film gas sensor deposited by screen-printing technique onto a micromachined hotplate. Sens. Actuators B Chem..

[69] Puigcorbé J., Vogel D., Michel B., Vilà A., Gràcia I., Cané C., Morante J.R. (2003). Thermal and mechanical analysis of micromachined gas sensors. J. Micromech. Microeng..

[70] Udrea F., Gardner J.W., Setiadi D., Covington J.A., Dogaru T., Lu C.C., Milne W.I. (2001). Design and simulations of SOI CMOS micro-hotplate gas sensors. Sens. Actuators B Chem..

[71] Gaiardo A., Demenev E., Bellutti P., Dolci C., Maestrini A., Antonelli F., Miotto V. (2020). New Chemoresistive Gas Sensor Arrays for Outdoor Air Quality Monitoring: A Combined R&D and Outreach Activities. ECS Meeting Abstracts.

[72] Suárez J.I., Arroyo P., Lozano J., Herrero J.L., Padilla M. (2018). Bluetooth gas sensing module combined with smartphones for air quality monitoring. Chemosphere.

[73] Wang S., Jiang Y., Tai H., Liu B., Duan Z., Yuan Z., Pan H., Xie G., Du X., Su Y. (2019). An integrated flexible self-powered wearable respiration sensor. Nano Energy.

